# Faster ciguatoxin extraction methods for toxicity screening

**DOI:** 10.1038/s41598-024-72708-1

**Published:** 2024-09-17

**Authors:** Christopher R. Loeffler, Astrid Spielmeyer

**Affiliations:** Department of Safety in the Food Chain, National Reference Laboratory for the Monitoring of Marine Biotoxins, German Federal Institute for Risk Assessment, Max-Dohrn-Str. 8-10, 10589 Berlin, Germany

**Keywords:** (max. 6, American spelling): marine biotoxins, Ciguatera, Neuro-2a bioassay, Mass spectrometry, Fish poisoning, Extraction, Biochemistry, Natural hazards

## Abstract

Ciguatera poisoning (CP) is a severe global public health problem caused by the consumption of seafood products contaminated with ciguatoxins (CTXs). The growing demand for seafood products requires high-throughput testing for CTX-susceptible seafood, however complex extraction and slow cleanup methods inhibit this goal. Herein, several methods for extracting CTXs from fish tissue were established and compared; these methods are sensitive, specific, and valid while achieving higher sample extraction throughput than currently established protocols. The trial fish material was generated from multiple species, with different physical conditions (wet and freeze-dried tissue), and naturally contaminated with various CTXs (i.e., CTX-1B, CTX-3C, and C-CTX-1), thus ensuring these methods are robust and broadly applicable. The extraction methods used were based on mechanical maceration with acetone or methanol or enzymatic digestion followed by acetone and ethyl acetate extraction. Crude extracts were investigated for CTX-like toxicity using an in vitro mouse neuroblastoma (N2a) cell-based assay (CBA). Among the three methods, there was no significant difference in toxin estimates (*p* = 0.219, two-way ANOVA), indicating their interchangeability. For speed (> 16 samples/day), accuracy (100%), and CTX analog retention confirmation by liquid chromatography-tandem mass spectrometry (LC‒MS/MS), the preferred extraction methods were both methanol and enzyme-based. All extraction methods post hoc confirmation of CTX analogs successfully met international seafood market-based CTX contaminant guidance. These methods can drastically increase global CTX screening capabilities and subsequently relieve sample processing bottlenecks, inhibiting environmental and human health-based CTX analysis.

**Supplementary Information:**

The online version contains supplementary material available at 10.1038/s41598-024-72708-1.

## Introduction

Ciguatoxins (CTXs) are a class of polycyclic polyether marine biotoxins produced by benthic dinoflagellates in the cosmopolitan genus *Gambierdiscus*^1^. While not all compounds produced by *Gambierdiscus* spp. have been fully elucidated, approximately thirty CTX isoforms have been described^1^. Previously, CTX-analogs were considered region specific, being produced by isolated species of *Gambierdiscus*; however, in light of invasive species movements^2,3^, climate change adaptations, and the international seafood trade^4–8^, seafood consumers can potentially be exposed to any CTX-analog. This potential for exposure, necessitates a globally applicable response protocol for the rapid detection and analysis of CTXs that accounts for a broad range of known and unknown CTX-analogs.

CTXs can accumulate (irrespective of the CTX-analog) in marine organisms feeding in shallow marine habitats where CTX-producing *Gambierdiscus* spp. are found^1,9–14^. CTXs have lipophilic properties and when ingested, may be metabolized and stored in various animal tissues, such as the meat (flesh), head, liver, viscera, and roe (eggs)^10,15–17^. Higher CTX accumulation in contaminated organisms have been associated with larger animal size and increased age. However, the severity of CTX endemicity in the region and the diet of the individual animal play significant roles in CTX accumulation. Because CTXs can accumulate (more or less) indiscriminately among ecological pathways, more than 425 fish species have been implicated as vectors for CTXs. Several genera, representing major commercially important marine fisheries resources, are frequently associated with CTXs, and these genera are regionally considered CTX-sentinel fish and include barracuda (Sphyraenidae), amberjack (Carangidae), grouper (Serranidae), snapper (Lutjanidae), and parrotfish (Scaridae)^1^. CTX-sentinal fish typically inhabit shallow marine habitats where CTXs are known to accumulate and either feed directly on algae where *Gambierdiscus* grows (i.e., herbivores), or are piscivorous fish feeding on animals contaminated with CTXs. Therefore, seafood safety managers use broad market guidelines that are designed to prevent CTXs from entering the commercial market, by restricting known CTX vectors or products from CTX endemic regions^18^. In emerging endemic regions like Macaronesia (Canary Islands (Spain), Azores, and Madeira (both Portugal) and Cabo Verde), management plans for CTXs are proactive. Guidance on species, location, and permitted animal size is provided to fish harvesters and a ‘first point of sale’ investigation for CTXs is conducted before a fish, that is subject to regulation, goes to the market. This control measure increased consumer food safety, allowing 7,717 (87%) large fishes to reach the commercial seafood chain in the Canary Islands with confirmed food security^19^.

When consumed, CTXs can cause gastrointestinal, cardiovascular^20^, central and peripheral neurologic symptoms in humans, resulting in ciguatera poisoning (CP)^21,22^. CP is a burden for those afflicted^23,24^ and global incidence rates are estimated between 10,000 and 50,000 cases per year^1^. Seafood processors linked to outbreaks of CP have faced international enquiries and export restrictions^4^, which can disrupt the wider global fisheries food supply chain^25^. Recently, CP has had a more prominent global reach, occurring in nonendemic regions (e.g., Germany), requiring local organizations to respond to outbreaks and develop costly and difficult to implement monitoring or response programs^5,7^. International guidelines have been developed by working groups (i.e., FAO, EFSA, CCCF, FDA-HAACPT) providing examples of how to address the complexities of CP, to prevent, reduce, and respond to CP outbreaks^1,18,26^. However, current testing protocols for detecting CTXs, prior to consumption, were not designed to meet global seafood demands. CTX testing at present, lacks a high capacity, and research on the topic in general is limited in scope. In 2022–2023, only 83 scientific articles were published on the topic of ciguatera (according to a search of ‘ciguatera’ in the ‘Article title, Abstract, and keywords’ using the Web of Science and Scopus); of these, only a small handful dealt with CTX analysis. CP is not under routine surveillance in the European Union and existing testing and control effort shortfalls highlight a substantial gap between annual CTX research capacity (~ 1000 fish), the incidence rate of CP, testing efforts to preempt CTXs from entering the market by seafood processors (unknown), and fisheries demand (> 10,000,000).

Testing protocols can reduce CP incidences^19^, but the extraction process is slow and complex^11,27,28^, creating a bottleneck for increasing testing. CTXs are potent in minute (< ppb) quantities and contained in complex matrices, and the sample extract must be semipurified before analysis either by the mouse bioassay, immunosensor^29^, fluorescent receptor binding assay^30^, a cell-based assay, or mass spectrometry. In contrast to extraction protocols, analytical and biological testing methods for CTXs are both fast (meeting high-throughput requirements) and accurate, where cell-based cytotoxicity assays are capable of analyzing 96-1536 samples in a 48-h period^31^. Thus, a fast, effective, and selective extraction method for separating CTXs from coextracted seafood matrices for analysis is urgently needed.

This study investigated three rapid CTX extraction methods designed to increase sample extraction throughput (> 80 samples week^−1^). The test material used included various fish species (*Enchelynassa canina*,* Sphyraena barracuda*,* Lutjanus bohar*, and *L. malabaricus*), with different water content conditions (wet or dry). The test material included all major CTX analogs (i.e., the CTX-4A and CTX-3C groups, C-CTX-1) and included a fish involved in a CP outbreak (*L. bohar*^4^) or fish from endemic CP regions. Together these fish were from the Pacific (*E. canina* from Hawai’i^10^), from an island in the Caribbean^9,32^ (*S. barracuda*^33^), and Indian Ocean. The crude extracts generated from the methods were tested for CTX-like activity using a bioassay designed to detect CTX-like cytotoxicity (Neuro-2a bioassay). CTX-analog profiles were investigated by LC‒MS/MS after undergoing further cleanup.

## Results and discussion

### CTX extraction

The species used herein are ‘sentinel species for CP’, the family Muraenidae is often implicated in CP^34,35^, and 29% of CP outbreaks from 1998 to 2015 in the United States were attributed to *S. barracuda* and *Lutjanus* spp^36^. In the event of a CP outbreak, a diagnosis is made by medical professionals based on the patients’ symptoms (e.g., neurological and gastrointestinal), along with a dietary history of consuming fish associated with CP within 24–48 h of presenting symptoms^37^. To confirm the CP diagnosis, a portion of the meal remnant must be tested and confirmed to contain CTXs^33^, or if not available, samples of the original product prepared for consumption should be investigated. To test for CTXs, current methods often require 10–120 g of fish tissue for analysis^10,27,38–40^. However, meal remnants or remaining products are often limited^41^; therefore, in this study, the extraction material was restricted to < 6 g of wet tissue or 1 g of dry tissue, ensuring its applicability in situations with severely limited meal remnant material. Freeze-dried material was included in the study to replicate the transfer of stable sample material between laboratories.

**Fig. 1:**
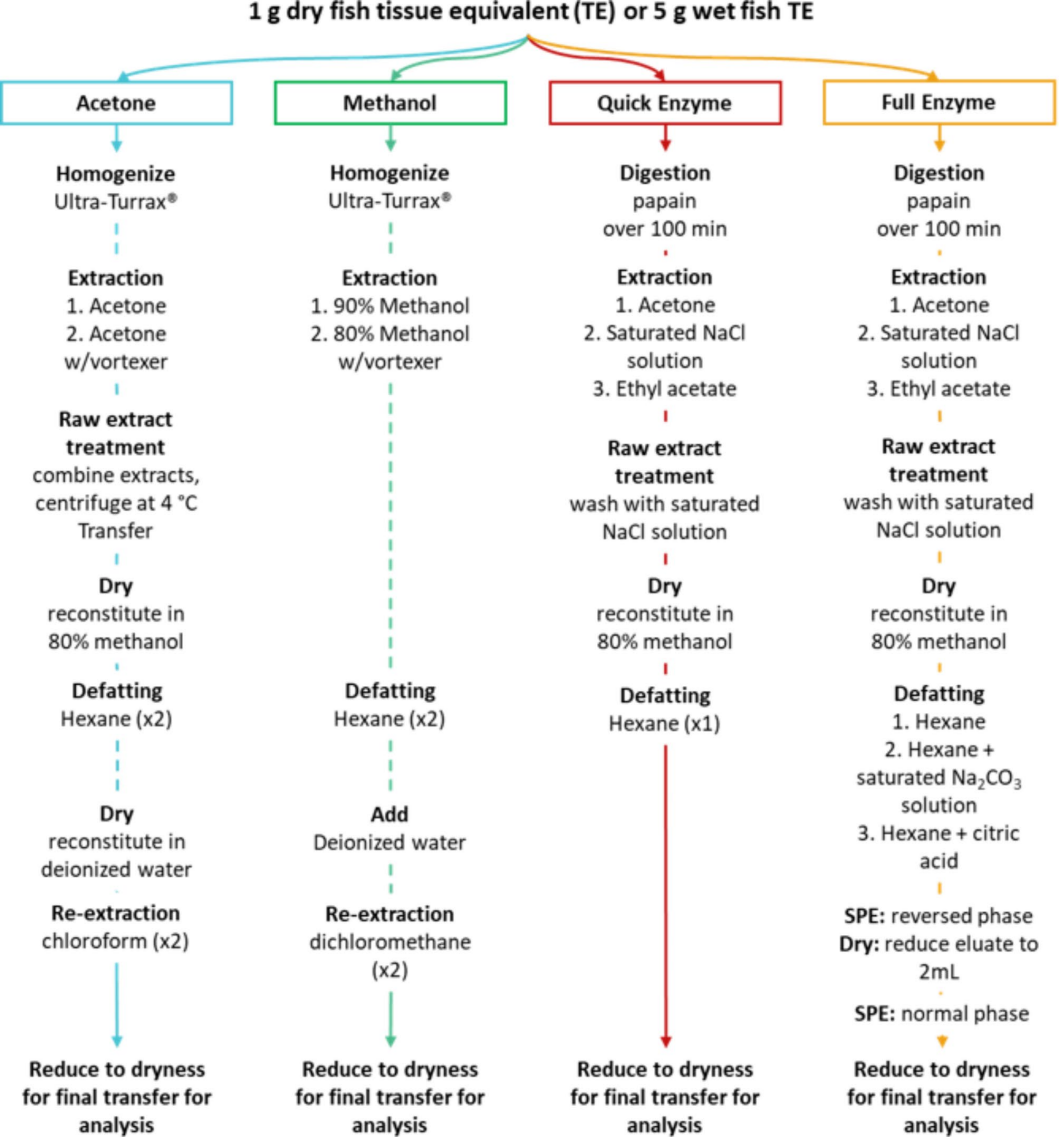
Flow diagram of the batch liquid‒liquid extraction and full clean-up methods. The acetone method was developed by Dickey^27^ and modified herein; the quick enzyme and full enzyme methods were developed by Spielmeyer et al.^45^. Fish tissue used was 1 g dry or 5 g wet tissue equivalent (eq.). Tissue to solvent volume ratios are described in the ‘Sample extraction’ section of the respective methods. Final residues were reconstituted in methanol for analysis.

Material mass and solvent volume are correlated, therefore, lowering the sample mass for extraction can reduce solvent usage. Reducing solvent usage has several advantages for cost, time, ease of handling, and reduces the environmental impact of CTX analysis^42^. Hazardous solvents contribute to ground-level ozone, ozone depletion, and groundwater pollution, while presenting direct risks of toxicity, exposure, and volatility^43^ to the user. Therefore, lower impact alternatives should be considered and used when possible. CTX extraction methods can involve ≥ 2–4 drying steps, and the acetone (ACT) method had the longest drying time among the methods tested in this study, due to drying extracts containing water and lipids. The methanol (MeOH) method was the highest throughput method tested, and was based on the ACT approach but modified with alternatives for acetone and chloroform, and the intermediate drying steps were eliminated (Fig. 1). Chloroform was replaced by dichloromethane (DCM) in the MeOH method because DCM has a lower anesthetic effect and a lower impact on the environmental degradation of the ozone layer^44^. Because CTXs are bound in tissue, the enzymatic digestion method^45^ was selected based on the general principle that a finer particle size is related to better extraction efficiency, allowing the penetration of solvents and diffusion of solutes. For both enzyme methods (quick enzyme, Q-Enz; full enzyme, F-Enz), no halogenated solvents were utilized.

Time is one of the most important factors for sample-throughput, and all the improved efficiency methods were capable of completing the extraction process for 16 samples on a working day, easing the extraction bottleneck for CTX analysis. Based on the laboratory capacity of the study, the total processing times for the extraction of 16 samples (limited to 16 by centrifuge rotor space) for each method were 9, 4.5, and 6.5 h for the ACT, MeOH, and Q-Enz methods, respectively. The MeOH method has the highest throughput, in part by drying only in the final step with a low volume of a volatile solvent (Table 1). The ACT, MeOH, and Q-Enz methods require 3–4 transfer steps (Fig. 1; Table 1), with 60% of these transfers occurring during subsequent re-extractions of the original sample material. The transfer steps used in a method can be prone to error, leading to material loss, and can be a slow processing point through increased handling time while also creating additional work for cleaning or disposable vessel waste. The Q-Enz method had the least total number of steps, eliminating most of the centrifugation and transfer processes compared to the other methods. Using centrifugation improved the phase separation; however, this can also be a numerically limiting factor restricted by centrifuge rotor space (as was the case in this study).

**Table 1:** Handling steps and average EC_50_ (mg tissue eq.) for each method.

Steps	Method			
	Acetone	Methanol	Quick-enzyme	Full-enzyme
Ultra Turrex	1	1	0	0
Cooking	0	0	1	1
EnzymATIC degradation	0	0	1	1
Vortex	5	5	6	8
Tube transfer	4	4	3	3
Centrifugation	7	7	3	5
Drying	3	1	2	4
Solid phase extraction	–	–	–	2
Total steps	20	18	16	24
Average EC_50_* (mean tissue eq. ± standard error of the mean)	0.101 ± 0.010 (*n* = 24)	0.091 ± 0.009 (*n* = 27)	0.077 ± 0.009 (*n* = 27)	0.128 ± 0.023 (*n* = 27)

*EC_50_ values averaged from 27 independent 96-well plates for 3 species and 3 replicates per species (except for Acetone, *n* = 24 plates; one sample was negative).

To reduce the extraction processing time, several optional steps were omitted. Raw fish tissue can undergo an initial cooking step to denature proteins^46^, but this step was omitted from the ACT and MeOH extraction methods. This step is part of the Q-Enz method, as enzymatic digestion requires a temperature of 60 °C; however, this is a ‘hands-off’ step that does not require the attention of the user. The greatest time, cost, and material savings were realized by eliminating, avoiding, or postponing solid-phase extraction (SPE) cleanup. Using a streamlined CTX screening sample throughput design, only extracts deemed ‘positive for CTX-like activity’ by CBA should undergo the SPE-based cleanup required for LC‒MS/MS analysis. SPE cleanup is effective at removing interfering matrix components; however, it is a time- and resource-intensive process that can be sensitive to errors. All three methods applied herein were tested without SPE cleanup by CBA, and the toxin amount was semi-quantified. SPE cleanup was only conducted on samples for CTX confirmation by LC‒MS/MS analysis.

### Toxin analysis by CBA

**Fig. 2:**
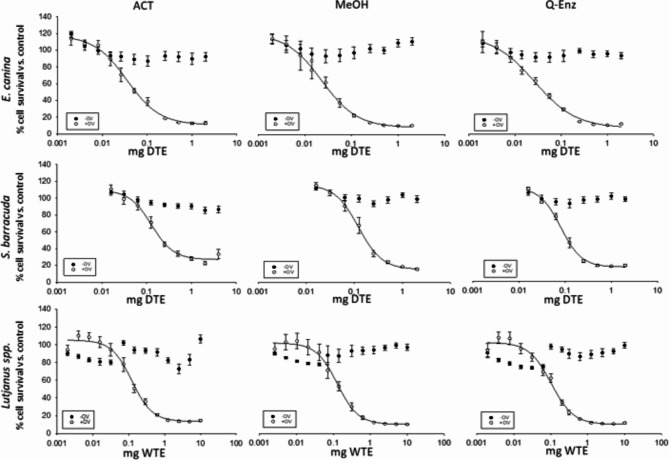
Combined concentration‒response curves from the cell-based assay derived without the addition of ouabain (O) or veratridine (V) (– OV, solid symbols) and with the addition of OV (+ OV, open symbols) when exposed to various concentrations of semipurified fish extracts. The cells were exposed to 0.22 and 0.022 mM O/V, respectively. Species are listed from top to bottom: *E. canina* (mg DTE), *S. barracuda* (mg DTE), and *Lutjanus* spp. (mg WTE). The extraction methods used were as follows (from left to right): acetone (ACT), methanol (MeOH), and quick-enzyme (Q-Enz). Each graph represents an average of *n* = 3 independent 96-well plates per extract (see Table 2 for individual results in the CTX-1B eq.). Error bars represent the standard deviation from all independent 96-well plate analyses performed for each sample (minimum of three independent 96-well plates; each assay included three replicate points).

The total range of fish tissue equivalent (TE) used was 0.002–10.0 mg of TE. No major inhibiting matrix effects were observed at the highest tested tissue matrix equivalent (10 mg Dry Tissue Equivalents (DTE) (Supplemental Fig. 1) or Wet TE (WTE)) (Fig. 2). Matrix interference effects were tested for each extraction method using eight blank (CTX-negative) fish samples (Supplemental Fig. 1). All the extracts for toxin analysis tested by the CBA herein were ‘positive’ for CTX-like activity (Fig. 2), except for one sample (*E. canina* ACT-3) (Table 2). The sample was confirmed to be ‘negative’ both by CBA and by LC‒MS/MS (i.e., a true analytical ‘CTX-negative’), but based on a priori sample information, this was considered a ‘false CTX-negative’. All the samples were prepared in parallel, and the other samples from the *E. canina* ACT triplicate (e.g., ACT-1 and - 2) were ‘CTX-positive’. Therefore, it was presumed that the CTX-analytes were lost during the extraction process for this replicate; however, the step where the CTX-analytes were lost remains unknown. Among all methods, the level of CTX accuracy was high (26/27), with a specificity of 98%. We can report high confidence according to the mode of action of the toxin in a functional assay format^47^. Among all the methods, the mean EC_50_ ± SDs for each species among all replicates were 0.116 ± 0.037 (*n* = 27), 0.122 ± 0.030 (*n* = 27), and 0.030 ± 0.005 (*n* = 24) for the *Lutjanus* spp., *S. barracuda*, and *E. canina* samples, respectively (Table 2). The EC_50_ for the CTX-1B standard, calculated from the dose‒response curve, was 0.183 ± 0.052 pg (*n* = 4), and the coefficient of variation (%CV) was 29% (Table 2). Among the analysis days and sample types (*Lutjanus* spp., *S. barracuda*, *and E. canina*), the %CV ranges for the ACT, MeOH, Q-Enz, and F-Enz extraction methods were 8–25, 11–18, 3–34, and 4–31%, respectively (Table 2), and similar to the CTX-1B standard (29%). The species with the least variability among the replicates was *E. canina*, which was the most toxic sample and required less material for analysis. *Lutjanus* spp. had the highest variability among replicates; this was the only fresh frozen tissue sample. Due to the limited availability of contaminated material, only 1 g of unhomogenized fish fillet tissue was used for extraction, which was combined with 4 g of noncontaminated fillet. This low sample size might have contributed to the greater variability. Furthermore, Lewis et al.^46^ found that variations in the water content of a sample can potentially influence extraction efficiency.

**Table 2:** CTX-1B toxin equivalency (ng CTX-1B eq. Per g tissue) in samples, based on the concentration‒response curves (EC_50_) measured from the CBA.

Species	Tissue replicate	ng CTX-1B eq. per g tissue			
		ACT	MeOH	Q-Enz	F-Enz
*E. canina*	1	5.545	6.310	6.100	3.050
	2	4.692	7.625	6.536	4.463
	3	–	8.318	5.903	2.080
	Avg.	5.119	7.418	6.180	3.198
	Std. Dev	0.427	0.833	0.264	0.979
	%CV	8%	11%	4%	31%
*S. barracuda*	1	1.307	1.307	2.011	2.288
	2	1.076	1.220	2.127	1.577
	3	1.525	1.664	2.153	1.428
	Avg.	1.303	1.397	2.097	1.779
	Std. Dev	0.183	0.192	0.062	0.359
	%CV	14%	14%	3%	20%
*Lutjanus* spp.	1	1.664	1.830	2.614	0.871
	2	2.033	1.220	2.033	0.832
	3	1.076	1.867	1.076	0.915
	Avg.	1.591	1.639	1.908	0.872
	Std. Dev	0.394	0.297	0.634	0.034
	%CV	25%	18%	34%	4%
		CTX-1B ng			
Standard	1	0.273			
	2	0.161			
	3	0.152			
	4	0.146			
	Avg.	0.183			
	Std. dev.	0.052			
	%CV	29%			

(–) Negative by CBA and LC‒MS/MS; all species (*E. canina*, *S. barracuda*, and *Lutjanus* spp.) and all extraction methods (acetone, methanol, quick-enzyme, and full-enzyme) are listed by average toxin equivalency (among independent sample extracts) (Avg.), standard deviation (Std. Dev.), and coefficient of variation (%CV) based on a minimum of three independent 96-well microplates per tissue replicate.

To investigate the effects of the extraction method (ACT, MeOH, Q-Enz), species, or any interaction between these factors on the toxins estimated from the sample, a two-way ANOVA was conducted, and the results revealed that there was no statistically significant interaction effect between the extraction method and species (F = 0.827, df = 4, *p* = 0.526). Simple main effects analysis revealed that the extraction method did not have a statistically significant effect on the toxin estimate (F = 1.661, df = 2, *p* = 0.219). The false negative sample *E. canina* ACT-3 was excluded from these tests. The results indicated that the three methods tested herein had relatively similar amounts of toxin that were recovered during the extraction process. However, a one-way ANOVA was performed to determine if there was a statistically significant difference between the extraction methods on the toxin estimation for each species. For *S. barracuda* the one-way ANOVA identified a significant difference among the methods applied (F = 6.781, df = 2, *p* = 0.029). A pairwise multiple comparison procedure (Tukey’s HSD Test) found that the mean value of the toxin estimate was significantly different between the ACT method and the Q-Enz method (*p* = 0.031). For *E. canina*, there was a significant difference among the methods applied (F = 7.466, df = 2, *p* = 0.032), and the ACT method was significantly different than the MeOH method (*p* = 0.027, Tukey’s HSD Test). No difference was found among the methods for *Lutjanus* spp. (F = 0.0745, df = 2, *p* = 0.929).

The average EC_50_ (mean ± standard error (SE)) in mg of TE for each extraction method and all matrices was 0.101 ± 0.010 for ACT, 0.091 ± 0.009 for MeOH, and 0.077 ± 0.009 for Q-Enz (Table 1), where lower values indicate less material was required to acquire the EC_50_. The ACT method had the highest tissue amount required for the generation of an EC_50_, indicating a lower toxicity and thus a lower extraction efficiency. Simple main effects analysis also revealed that ‘species’ was significantly related to the ‘toxicity estimate’ (F = 28.17, df = 2, *p* < 0.001). Post hoc comparisons among species revealed that *E. canina* vs. *S. barracuda* and *E. canina* vs. *Lutjanus* spp. were significantly different (*p* < 0.001), whereas *S. barracuda* versus *Lutjanus* spp. were not significantly different (*p* = 0.663). The species contained significantly different toxin concentrations and qualitatively different CTX profiles. Animals can contain varying quantities of naturally occurring CTXs as a consequence of their feeding and migratory behavior^9,48–51^.

Even among these qualitative and quantitative CTX differences, the extraction method used as intended among different CTX-analogs and tissue types, caused no significant difference (*p* > 0.01) in the total composite toxin estimates by CBA. Therefore, all extraction methods used herein can be considered ‘functionally similar’ among the conditions of variable CTX concentrations and CTX profiles. However, the ACT method had several drawbacks including a false negative result, the lowest extraction efficiency, and the longest extraction process. Therefore, while all methods are functionally similar, users should decide which method is best suited to their laboratory infrastructure and their individual CTX analysis goals.

**Fig. 3:**
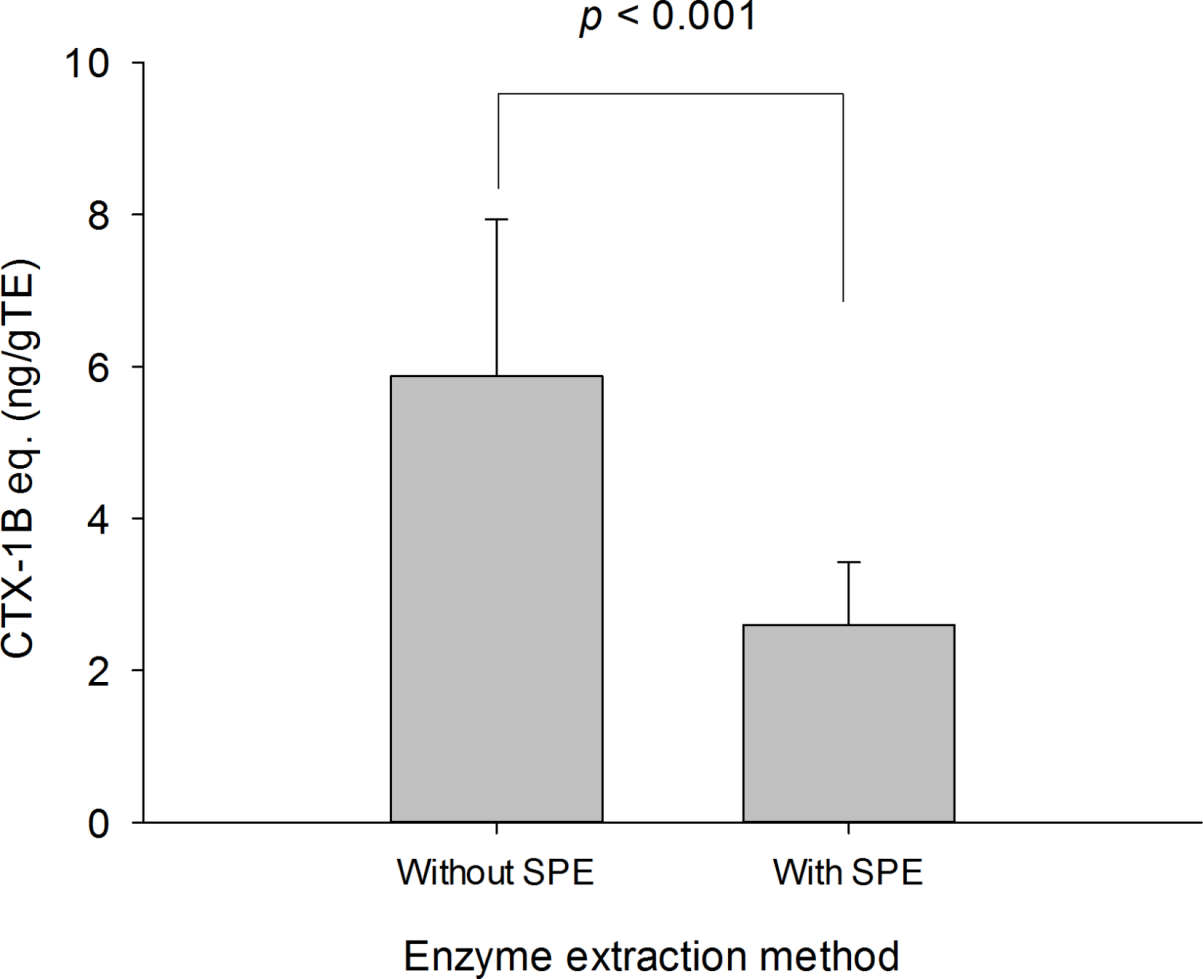
Comparison of the Q-Enz (without SPE) and F-Enz (with SPE) extraction methods for the nine fish sample extracts from three species (*E. canina*, *S. barracuda*, and *Lutjanus* spp.). The results represent ng CTX-1B eq. per g tissue eq. as determined by the cell-based assay; the *p* value refers to *t-*test results.

Fish tissue is a complex matrix, and because CTXs are found as trace contaminants (< 1 µg kg^−1^), their detection by LC‒MS/MS can become a difficult challenge. SPE is a commonly technique applied to extracts for removing unwanted matrix components. All fish species were extracted using the Q-Enz and F-Enz methods and thus tested before (Q-Enz) and after SPE (F-Enz). There was a statistically significant difference between the Q-Enz (pre-SPE) and F-Enz (post-SPE) samples based on the results of the *t-*test (*p* < 0.001) (Fig. 3). The mean ± SE of the Pre-SPE method (Q-Enz) was 3.37 ± 0.716 ng CTX-1B eq. g TE^−1,^ and the mean ± SE of the Pre-SPE method was 1.95 ± 0.401 ng CTX-1B eq. g TE^−1^, indicating a toxicity loss of approximately 40% in the samples subjected to SPE cleanup. Losses might occur during additional defatting steps under alkaline and acidic conditions. This procedure was performed according to Nagae et al.^52^, who reported recovery rates above 70% based on LC–MS/MS analysis for their entire sample preparation protocol. Additional losses might occur during the reversed-phase SPE cleanup step if analytes are not fully retained on the column material. Losses during normal-phase-SPE can be excluded, as both fractions (filtrate and eluate) were retained for further analyses. During the development of the F-Enz method, an average extraction efficiency of 68% was determined for all the analytes and matrices tested^45^. This finding implies that the Q-Enz method and the other methods tested herein recovered a high proportion of the CTX toxicity contained in a sample.

The CBA can be sensitive to matrix interferences and tests of maximal concentrations of wet weight fish tissue have been recommended from 10 to 50 mg TE well^−1^ (i.e., 43–217 mg TE mL^−1^)^53,54^. The cell line used herein was previously tested on 162 mg of WTE (i.e., 704 mg TE mL^−1^ or 0.005 ng CTX-3C g WTE^−1^) from *Lutjanus* spp. post SPE cleanup, and the extract had no adverse effects on the matrix^55^, whereas herein we used 10 mg TE well^−1^ (i.e., 0.0183 ppb CTX-1B eq.) or 43 mg TE mL^−1^ for unpurified extracts. In the context of what CTX values are relevant for CP (i.e., at which level symptoms might occur after consumption of the seafood product), the recommended maximal consumption level in Japan is 70 ng CTX-1B per 70 kg body weight of the consumer^56^ or 0.175 ng CTX-1B eq g^−1^ fish tissue^57,58^. In the United States, based on the ‘Fishery and Fishery Products Hazards and Controls Guidance’ (June 2022 edition^18^), a guidance level of ≥ 0.1 µg kg^−1^ for C-CTX-1 equivalents (eq.) and ≥ 0.01 µg kg^−1^ CTX-1B eq. is provided for seafood products (based on^27,41^); no guidance level has been established for Indian CTXs. The EU represents the world’s second largest seafood market, and products containing CTXs should not be placed on the market^59–61^. Therefore, the demonstrated semiquantification level achieved herein was ~ 0.02 µg kg^−1^ CTX-1B eq., ensuring the suitability of the methods for meeting or exceeding global guidance values.

To ensure that the functional analysis of CTXs can meet user testing demands, the CBA has a flexible, user-friendly layout. With respect to a single standard 96-well plate, an ultrahigh sample throughput can screen twenty-four samples (no replicates), a fast screening can test eight samples (with triplicates per sample, e.g., performed for samples in Supplementary Fig. 1), or a single sample can be analyzed semiquantitatively for CTX-like toxicity. At an ultrahigh throughput of twenty-four samples per plate, only each sample was screened for CTX-like activity in a +/– format, with single ‘yes’/’no’ CTX-like activity being determined for each sample. The fast screening at eight samples per plate provides a higher reliability, as triplicates were investigated per sample. In both cases, samples that were deemed ‘positive for CTX-like activity’ were then retested using a full dose‒response curve (e.g., Fig. 2). The maximum number of samples that can be screened or quantified is user defined based on their 96-well plate single-day completion factor (limited to *n* = 28 plates per day in this study). The sample throughput by number is also related to time, and the CBA (as currently designed), regardless of the sample number, is typically a 48-h (or 24-h^62^) method (e.g., plate seeding, sample dosing, and plate reading). The extraction method is historically recognized as the bottleneck for the CTX analysis process; however, the fast extraction methods demonstrated herein can exceed the CBA single-sample-per-plate layout sample analysis throughput of a single user (e.g., over 48-h; *n* = 32 samples extracted vs. *n* = 28 analyzed by the CBA).

### Evaluation of samples by LC–MS/MS

Within the two-tier approach of initially screening samples with a CBA and confirming CTX analogs by LC–MS/MS^41^, samples deemed ‘CTX-positive’ in the CBA are transferred to LC–MS/MS for analysis. Sample extracts require SPE cleanup to be suitable for LC-MS/MS analysis; thus, all three fast extraction methods underwent the cleanup procedure, as described for the F-Enz method, starting with defatting under alkaline and acidic conditions, followed by two orthogonal SPE cleanup steps (Fig. 1). As only one congener (C-CTX-1) was detected in the F-Enz samples of *S. barracuda* (for more information on this sample see^63,64^), full cleanup was performed only for fast extracts of *E. canina* and *Lutjanus* spp. to check the success of the cleanup protocol on matrix compound removal as well as the effect of the initial extraction on the CTX profile. The results were compared with those obtained by the F-Enz method.

**Fig. 4:**
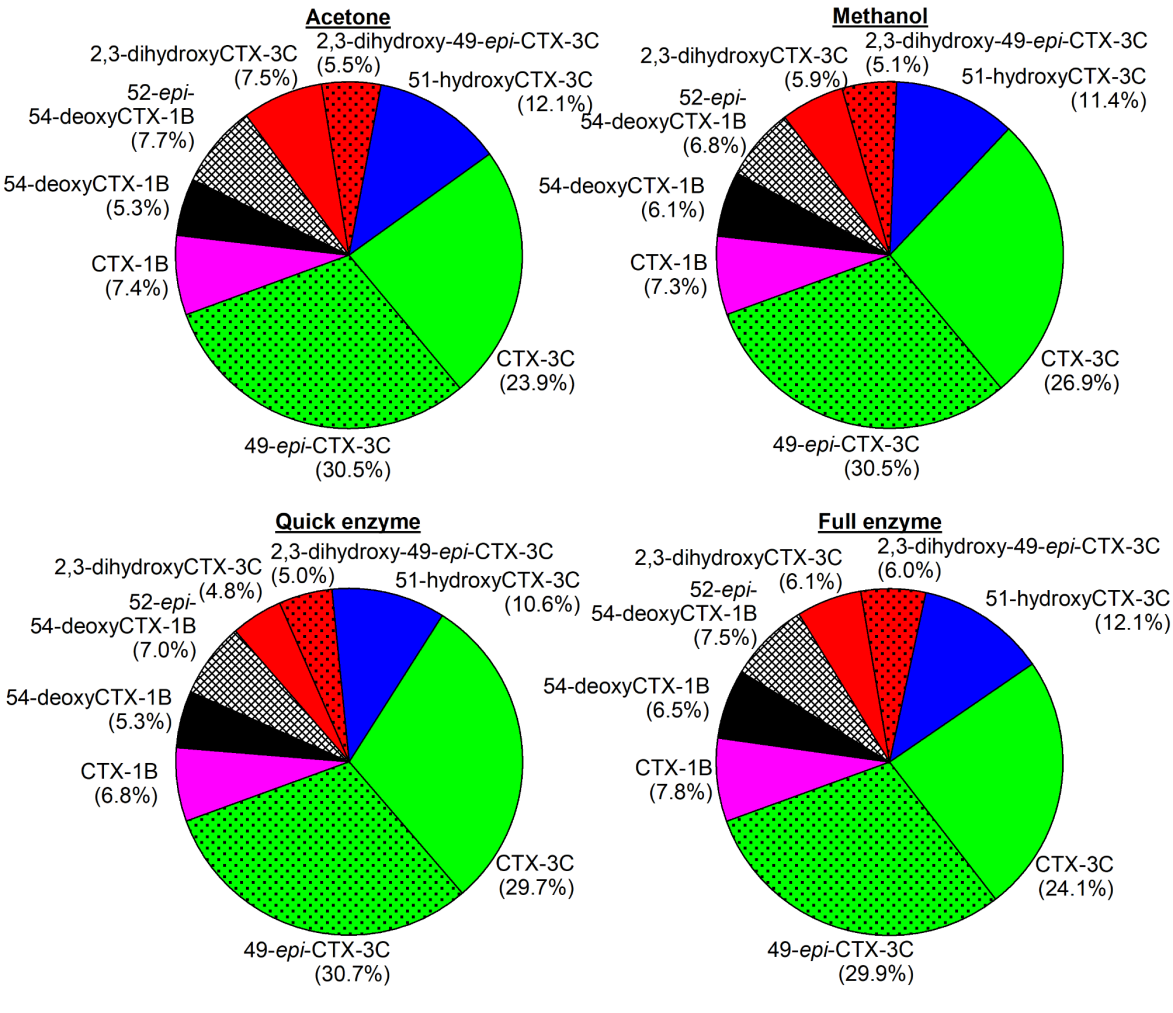
CTX profiles from *E. canina* after full cleanup of the fast method’s extracts for the acetone, methanol, and quick-enzyme methods compared to the profile of the full-enzyme extraction method; the results of the LC–MS/MS analysis are presented as percentage values based on the average proportion of compounds among the samples according to extraction method and species (*n* = 3, except acetone, here *n* = 2).

Differences were observed concerning the obtained extract purity for the respective methods and tissue types utilized. While all extracts showed comparable baseline intensities in case of the freeze-dried *E. canina* sample, wet-tissue sample extracts of the *Lutjanus* spp. from the ACT method revealed a higher baseline in the LC‒MS/MS chromatograms than samples from the other protocols (Supplemental Figs. 2 and 3). These findings indicate that compounds extracted by the ACT method in the first step could not be efficiently removed from the extract by the cleanup protocol utilized in this study and that samples extracted via the ACT method might require a different approach. Nevertheless, CTX analogs could be detected in those extracts as well, though with a reduced intensity compared to that of the MeOH and Q-Enz methods (Supplemental Figs. 2 and 3). One exception was sample #3 of *E*. *canina* (ACT method). This sample had a negative result for CBA (Table 2), and no CTX analogs were detected by LC‒MS/MS accordingly (Supplemental Fig. 4). For extracts of *E. canina*, similar profiles were obtained by all three primary extraction methods (Fig. 4). This finding suggested that CTX analogs can be extracted from the fillet tissue with comparable efficiencies independent of the utilized solvent. However, the extraction profiles might differ between the raw extracts without SPE cleanup^65^, and the application of SPE might lead to uniform profiles in the final extracts. This point could not be clarified because raw extracts are not suitable for reliable LC–MS/MS analysis.

**Fig. 5:**
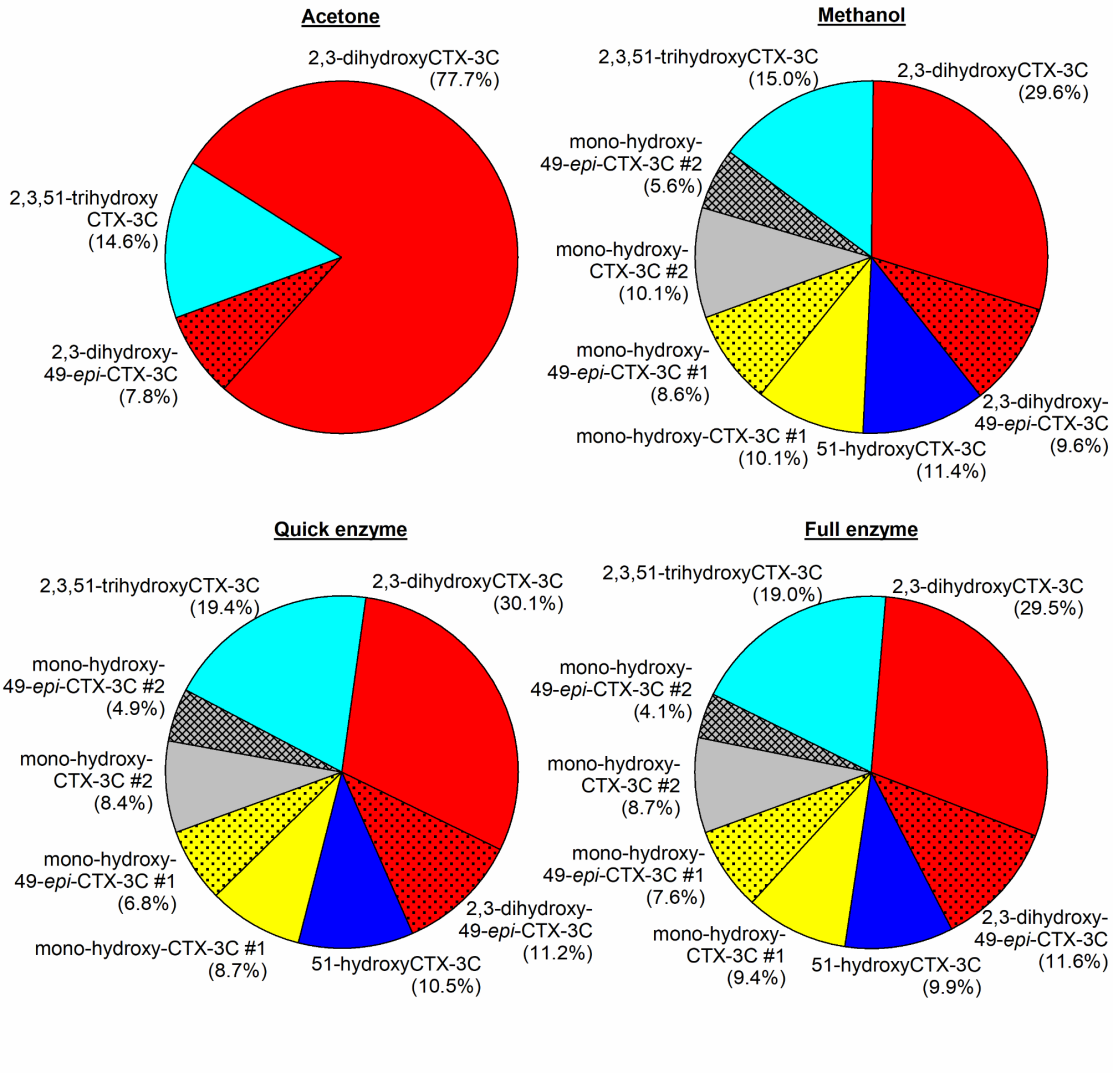
CTX profiles from *Lutjanus* spp. after full cleanup of the fast method’s extracts for the acetone, methanol, and quick-enzme methods compared to the profile of the full-enzyme extraction method; the results of the LC‒MS/MS analysis are presented as percentages based on the average proportion of compounds among the samples according to the extraction method and species (*n* = 3).

In the case of *Lutjanus* spp., the profiles differed among the extraction methods. For the ACT method, only three analogs could be identified, whereas the other methods revealed peaks of potentially eight analogs (Fig. 5, Supplemental Fig. 3). This is attributed to the lower extract purity (see above) which hampers the detection of the lower concentrated CTX-analogs. The purpose of confirming CTX analogs during the CTX analytical process is only qualitative confirmation of the CTX analogs in a sample. Therefore, the extracts obtained by the fast extraction methods investigated in this study are suitable for this purpose and can thus be utilized for both analytical and biological detection (i.e., samples can be reinvestigated by CBA after further cleanup for LC–MS/MS).

## Conclusion

Ciguatera poisoning is a major global health issue; however, preventing CP through CTX contaminant testing requires screening commercial fish at a scale that cannot keep pace with seafood consumption demand. Currently, the main impediment to faster CTX testing is the extraction process. To remove this bottleneck, several extraction methods were tested, compared, and demonstrated to rapidly extract CTXs for testing at human health-relevant concentrations. All CTX extraction methods had similar CTX-like activity, as determined by a cell-based toxicity assay, with good reproducibility among tissue extract replicates. Expediting the extraction process was achieved by minimizing the solvent handling volumes and drying times by reducing the sample material. Together, these methods are broadly applicable and suitable for low meal remnant material availability, various species, tissue water content, and CTX profile analysis by LC‒MS/MS after further cleanup and for accommodating diverse laboratory infrastructures. To answer environmental questions about CTXs as natural contaminants in the food chain, an increase in spatial and temporal sample coverage is needed, and these methods enable the necessary increase in sample throughput volume. A higher sample throughput can allow broader coverage of CTX-check screening for seafood products and thus can help prevent outbreaks of CP and ensure a safe and secure food supply, meeting a major stated goal of global seafood safety organizations and food processors. The higher throughput CTX extraction methods demonstrated herein increase the ability of laboratories to screen more seafood products at a faster rate while retaining CTX profile accuracy and CTX-like toxicity assessments, which can increase the global capacity for CTX screening and CP analysis. The establishment of simple methods with the capability of higher sample throughput provided herein is an important and necessary advancement toward expanding the coverage of species and regions under surveillance for CTXs. Increased surveillance and coverage can shed light on the seeming sporadic nature of CP and provide a greater proportion of the public with a measure of protection against CP.

## Materials and methods

### Sample material

For the method comparison, a fish tissue matrix was used from four species (common names from FishBase^66^): *E. canina* (Longfang moray), *S. barracuda* (Great barracuda), *L. bohar* (Two-spot red snapper), and *L. malabaricus* (Malabar blood snapper), representing three distinct orders (Perciformes, Istiophoriformes, and Anguilliformes) that have been implicated in CP outbreaks. *S. barracuda* was harvested, gifted, and stored as freeze-dried material from the Food and Agriculture Organization (FAO) major fishing area 31 of the (Western Central) Atlantic Ocean (St. Thomas, United States Virgin Islands). *E. canina* was collected, gifted, and stored as freeze-dried material from the FAO major fishing area 77 of the Eastern Central Pacific Ocean (west coast of Hawai’i, United States of America). *L. bohar* was recovered as frozen (wet) material from commercial products from FAO major fishing area 51 of the (western) Indian Ocean (Kerala, India). *L. malabaricus* (wet material) was purchased from a wholesale market and originated from FAO major fishing area 71 in the (western central) Pacific Ocean. Specimens were previously authenticated as *L. bohar*^4^, *S. barracuda* and *E. canina*^63^ and tested for toxin suitability^4,63,64^. All experiments were performed in accordance with relevant guidelines and regulations.

### Sample extraction

For the freeze-dried samples (i.e., *E. canina* and *S. barracuda*), muscle tissue was pulverized into a powder mixed for homogeneity and a 1 g DTE sub-sample was used for each replicate. Dried sample tissue was rehydrated for at least 60 min before sample preparation (methods ACT and MeOH) or immediately before incubation in a water bath (methods Q-Enz and F-Enz) using 4 mL of deionized water (5 g rehydrated weight). For the wet tissue, 1 g of CTX-contaminated *L. bohar* was sub-sampled and mixed with 4 g of *L. malabaricus* (5 g of total WTE). For the latter, no CTX-like toxicity was detected in the CBA. Two fish (one toxic, one not) were needed to supplement a limited supply of contaminated fish material. With regard to the sub-sampling from the toxic sample, previous investigations have determined that flesh samples can be arbitrarily taken from the same filet for toxin analysis^15^.

All samples were prepared in triplicate for each method. Reduction steps were performed in a stream of nitrogen at 40 °C. For the ACT, MeOH, and Q-Enz methods, the final dried residue was reconstituted in 1 mL of methanol, which equated to 1 g of DTE per mL or 5 g of WTE per mL in the final extract. For the F-Enz method, the final dried residue was reconstituted in 0.5 mL of methanol (2 g of DTE and 10 g of WTE per mL in the final extract). In all the cases, the extract was transferred to a glass vial and stored at –20 °C. Centrifugation steps were performed at 1900 or 2200 *g* (depending on the sample vessel) for 3–5 min (depending on the solid residue separation). Vortexing steps were performed for 30 s in each case. To test the extraction methods for matrix effects, eight blank (CTX-negative) samples were extracted according to each method and tested by CBA at a maximum of 10 mg DTE or WTE.

### Method – “Acetone” (ACT)

The acetone (ACT) method is a modified version of the procedure described by Dickey (2008)^67^. The sample material was weighed into a 50 mL polypropylene (PP) tube. Then, 10 mL of acetone was added (2 mL per g rehydrated DTE or WTE), and the sample was homogenized for 1 min (ultraturrax at 10,000 rpm). The sample was centrifuged, and the supernatant was transferred to a new PP tube. The solid residue was resuspended in 10 mL of acetone (2 mL per g rehydrated DTE or WTE), vortexed, and centrifuged. The supernatants were combined and centrifuged at 4 °C to remove any remaining solid residue. The supernatant was transferred to a glass vessel and reduced to dryness. The residue was reconstituted in 5 mL of 80% aqueous methanol (*v/v*) and defatted twice with 5 mL of *n*-hexane (1 mL per g rehydrated DTE or WTE). The *n*-hexane phases were discarded, and the methanol phase was reduced to dryness. The residue was resuspended in 5 mL of deionized water (1 mL per g rehydrated DTE or WTE), and the CTXs were extracted twice with 5 mL of chloroform (1 mL per g rehydrated DTE or WTE). The organic phases were combined and reduced to dryness to obtain the final dried residue.

### Method – “Methanol” (MeOH)

For the methanol (MeOH) method, the sample material was weighed into a 50 mL PP tube. Then, 10 mL of 90% aqueous methanol (*v/v*) was added (2 mL per g rehydrated DTE or WTE), and the sample was homogenized for 1 min (ultraturrax at 10,000 rpm). The sample was centrifuged, and the supernatant was transferred to a PP tube. The solid residue was resuspended in 10 mL of 80% aqueous methanol (*v/v*) (2 mL per g rehydrated DTE or WTE), vortexed, and centrifuged. The supernatants were combined and centrifuged to remove any remaining solid residue, after which the supernatant was transferred to a glass tube. The extract was defatted twice with 5 mL of *n*-hexane (1mL per g rehydrated DTE or WTE), after which the hexane phases were discarded. The defatted aqueous methanol phase was diluted with 5 mL of deionized water. CTXs were extracted twice with 5 mL of dichloromethane (DCM) (1 mL per g rehydrated DTE or WTE). The DCM phases were combined and reduced to dryness to obtain the final dried residue.

### Method – “Quick-enzyme” (Q-Enz)

The quick enzyme (Q-Enz) method covers the first sample preparation steps of Spielmeyer et al.^45^. The sample material was weighed into a 50 mL glass tube. The sample was incubated for 15 min in a water bath at 60 °C. After the addition of 1 mL of papain solution (10 mg mL^−1^), the tissue was enzymatically degraded for 100 min at 60 °C and vortexing after 25, 50, and 75-min. The degraded sample was extracted using 7.5 mL of acetone, 2.5 mL of saturated sodium chloride solution, and 7.5 mL of ethyl acetate (vortex step after each solvent addition) (in total: 3 mL organic solvent per g rehydrated DTE or WTE). The sample was centrifuged, and the supernatant was washed with 1.5 mL of saturated sodium chloride solution, followed by another step of centrifugation. The organic layer was transferred into a glass tube and reduced to dryness. The residue was reconstituted in 5 mL of 80% aqueous methanol (*v/v*) and defatted with 5 mL of *n*-hexane (1 mL per g rehydrated DTE or WTE). The hexane phase was discarded, and the methanol phase was reduced to dryness to obtain the final dried residue.

### Method – “Full-enzyme” (F-Enz)

A detailed description of the full-length enzyme (F-Enz) method was provided by Spielmeyer et al.^45^. The first steps of sample preparation were conducted as described for the Q-Enz method (see above). After the first defatting step with 5 mL of *n*-hexane, the sample was not reduced to dryness, but two additional steps were conducted using 3.5 mL of *n*-hexane after the addition of 70 µL of saturated sodium carbonate solution, followed by 7.0 mL of *n*-hexane after the addition of 350 µL of 5% citric acid solution (0.7- and 1.4-mL *n*-hexane per g rehydrated DTE or WTE). The hexane layers were discarded after each step. The aqueous methanol phase was applied to a preconditioned reversed-phase SPE cartridge (Chromabond EASY, 3 mL, 200 mg; Macherey-Nagel, Düren, Germany). Elution was conducted with 3 mL of acetonitrile and 5 mL of acidified ethyl acetate (ethyl acetate + 0.1 vol% acetic acid). The eluate was reduced to 2 mL in a stream of nitrogen, diluted with 2 mL of *n*-hexane and applied to a preconditioned normal-phase SPE cartridge (Bond Elut Si (silica), 3 mL, 500 mg; Agilent, Waldbronn, Germany). After the sample was applied, 3 mL of *n*-hexane/acidified ethyl acetate (1:1, *v/v*) was added to the cartridge. All the volumes were combined with the ‘filtrate’ fraction. The ‘eluate’ fraction was obtained by applying 3 mL of acidified ethyl acetate and 7 mL of acidified ethyl acetate/methanol (3:1, *v/v*). Both fractions were reduced to dryness to obtain the final dried residues.

### Sample cleanup for LC‒MS/MS

To compare the CTX profiles in the respective extracts, samples obtained by the ACT, MeOH, and Q-Enz methods were further cleaned for analysis by LC‒MS/MS. An aliquot of 500 µL of the sample obtained by the respective method was reduced to dryness and reconstituted in 5 mL of 80% aqueous methanol (*v/v*). For cleanup, the procedure described in Section “Method – “Full-enzyme” (F-Enz)” was conducted, starting with the addition of 70 µL of saturated sodium carbonate solution, followed by defattening and SPE. The final fractions (filtrate and eluate) were reconstituted in 0.5 mL of methanol.

Sample analysis was performed according to Spielmeyer et al.^45^. using a low-resolution Sciex QTrap 6500 + tandem mass spectrometer (Sciex, Darmstadt, Germany) coupled to an Agilent 1290 Infinity II UHPLC system (Agilent, Waldbronn, Germany). Due to the low concentration of the individual CTX analogs, the number of monitored ion transitions was reduced to five for both sample sets based on previous analyses. The monitored analogs included sodium adducts ([M + Na]^+^) of CTX-1B, 54-deoxyCTX-1B and its 52-epimer; CTX-3C and its 49-epimer; 2,3-dihydroxyCTX-3C and its 49-epimer; and 51-hydroxyCTX-3C in the case of the *E. canina* sample. CTX-3C and its 49-epimer 2,3-dihydroxyCTX-3C and its 49-epimer, mono-hydroxy-CTX-3C analogs (e.g., putative 2-hydroxyCTX-3C) and their 49-epimers, 51-hydroxyCTX-3C and 2,3,51-trihydroxyCTX-3C, were monitored for the *L. bohar*/*L. malabaricus* samples. Gradient elution using an eluent system of (A) 1 mM ammonium acetate and 0.5% formic acid and (B) methanol/acetonitrile (3:1, *v/v*) was performed on a reversed-phase column (Gemini NX-C18; 150 × 2 mm, 3 μm; Phenomenex, Aschaffenburg, Germany). Details concerning the LC and MS parameters are provided in Spielmeyer, et al.^45^.

### Neuro-2a cell bioassay (CBA)

Extraction efficiency and extraction purity were tested using N2a-CBA. Mouse (*Mus musculus*) neuroblastoma (Neuro-2a) cells from the American Type Culture Collection (N2a CCL-131™) were purchased from LGC Standards GmbH (Wesel, Germany) LOT #63649750. The cells were maintained and treated according to published protocols^47,67,68^. The desensitization of the cells to ouabain (O) and veratridine (V) was performed according to Loeffler et al.^55^. The cells were incubated for 22–24 h after sample dosage. Cell viability was evaluated via the MTT colorimetric assay (3-(4,5-dimethylthiazol-2-yl)-2,5-diphenyltetrazolium bromide (MTT)) as previously described^68^ by measuring the intensity of formazan color development in treated and untreated wells, calculated and represented as a percentage of cell survival versus controls. The concentration of extract required to reduce cell viability by 50% (EC_50_) was determined in the sensitized cells, and the results were compared with those of a CTX-1B standard (purchased from Professor R. J. Lewis, The Queensland University, Australia, prepared in November 2005). A four-parameter logistic equation using SigmaPlot v 14.0 (http://www.sigmaplot.co.uk/splot/products/sigmaplot/productuses/prod-uses43.php) was used to define the EC_50_. Controls, standards, and sample dilutions were analyzed in triplicate within 96-well plates and among days. Toxicity estimates for samples were based on comparisons with plates dosed with the CTX-1B standard and calculated as (standard EC_50_/Sample EC_50_) and reported as CTX-1B equivalents (eq.) per sample unit (i.e., CTX-1B eq. per g tissue equivalent either on a wet or dry basis) as previously described^27,55,69^. Results were expressed in dry toxin standard weight per ml (e.g., pg mL^−1^) or fish TE (wet or dry) per mL (e.g., mg TE mL^−1^). For samples prepared according to the “Methods – “Full-enzyme” (F-Enz)” section, equal volumes of the filtrate and eluate were combined for analysis.

### Statistical analysis

A two-way analysis of variance (ANOVA) model was used to investigate the effects of the two main treatments, extraction method and species, and their interactions on the toxin estimates by CBA. Post hoc comparisons were performed using the Holm‒Sidak test. A one-way ANOVA was performed to compare the effect of the fast extraction methods (ACT, MEOH, Q-Enz) for the respective species on the toxin estimated by the CBA. Post hoc test for multiple comparisons were performed using the Tukey’s HSD Test. Data in both cases were first tested for homogeneity of variance and normality. A general difference between the enzyme methods with- and without-SPE cleanup was observed and a *t-*test was performed to determine whether the SPE treatment had an effect on the toxicity estimate. The data met the assumptions of a *t-*test. Statistical analyses were performed using SigmaPlot software (Ver 14.0 Systat Software, Inc., San Jose, California).

### Ethical statement

The four fish species used in this study are edible economic animals and were obtained from private individuals or the European Union commercial seafood market. After euthanasia, muscle tissue was obtained for experiments. Ethical approval was not needed.

## Electronic supplementary material

Below is the link to the electronic supplementary material.

Supplementary Material 1

## Data Availability

The datasets used and/or analysed during the current study available from the corresponding author on reasonable request.
