# Supplementary material for: Faster ciguatoxin extraction methods for toxicity screening
Source: Sci Rep. 2024 Sep 17;14:21715. doi: 10.1038/s41598-024-72708-1 (PMC11408646; doi:10.1038/s41598-024-72708-1)
Supplement: Supplementary file 1 — Supplementary Material 1 [file 41598_2024_72708_MOESM1_ESM.docx]

**Supplementary information**

**Faster c****iguatoxin extraction methods for toxicity screening**

Christopher R. Loeffler*^§^, Astrid Spielmeyer^§^

German Federal Institute for Risk Assessment, Department of Safety in the Food Chain, National Reference Laboratory for the Monitoring of Marine Biotoxins, Max-Dohrn-Str. 8-10, 10589 Berlin, Germany

*Corresponding author: Christopher.loeffler@bfr.bund.de

^§^Contributed equally to the work


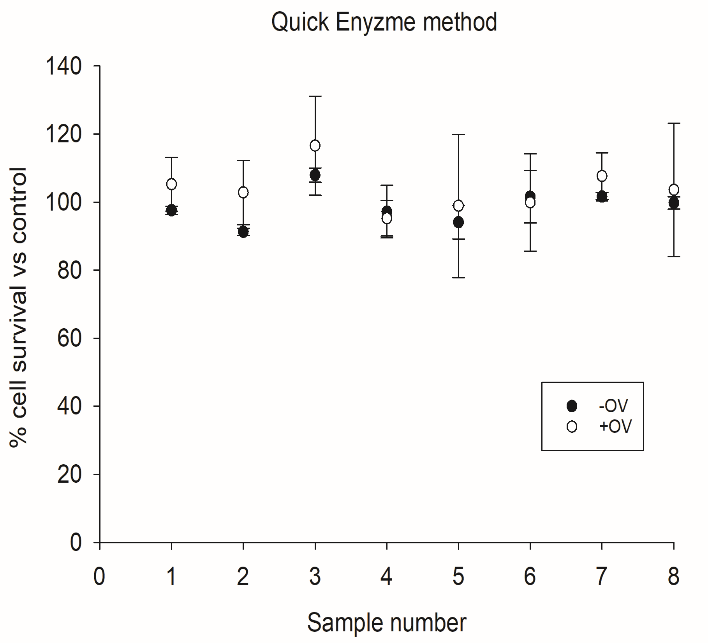

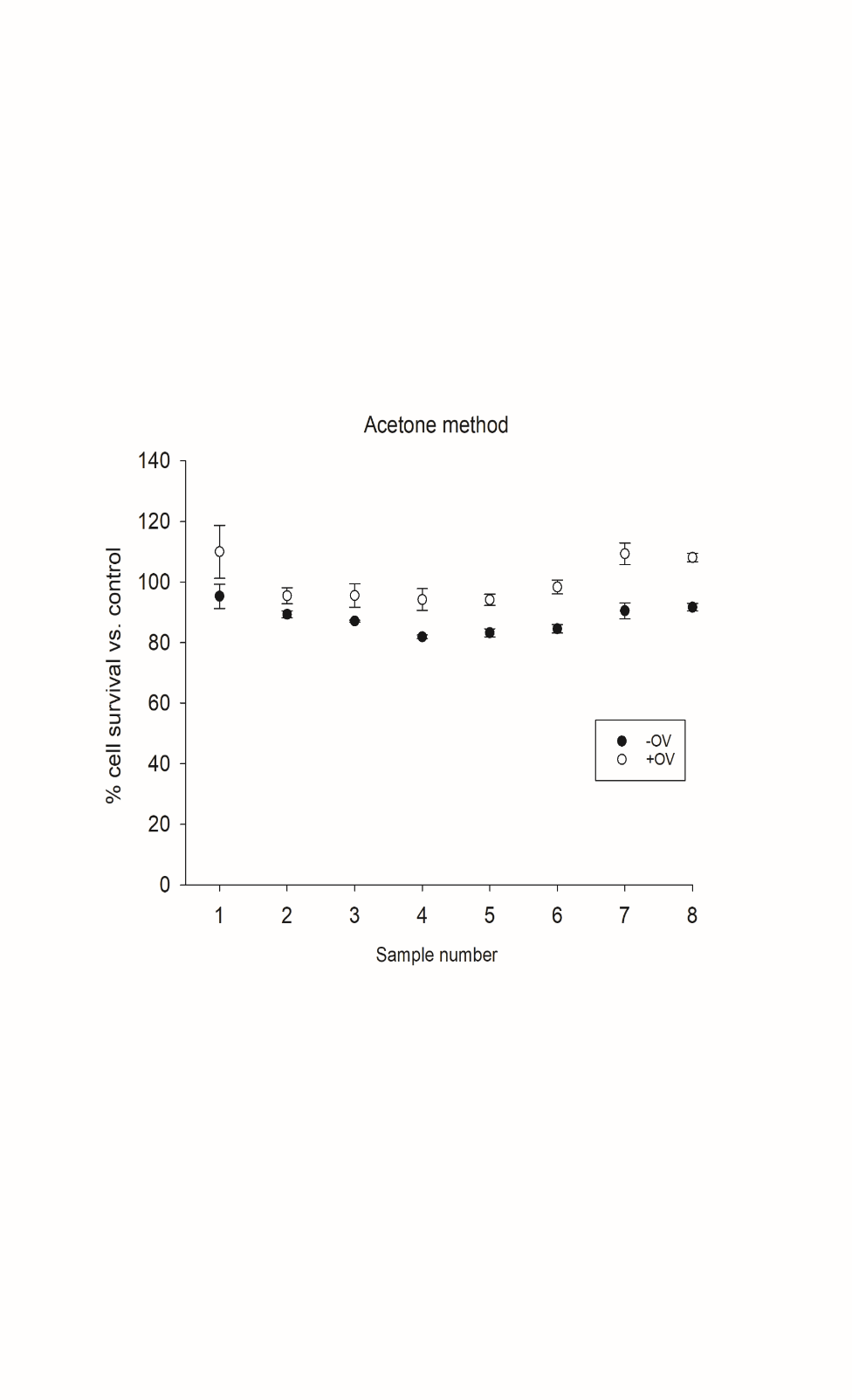

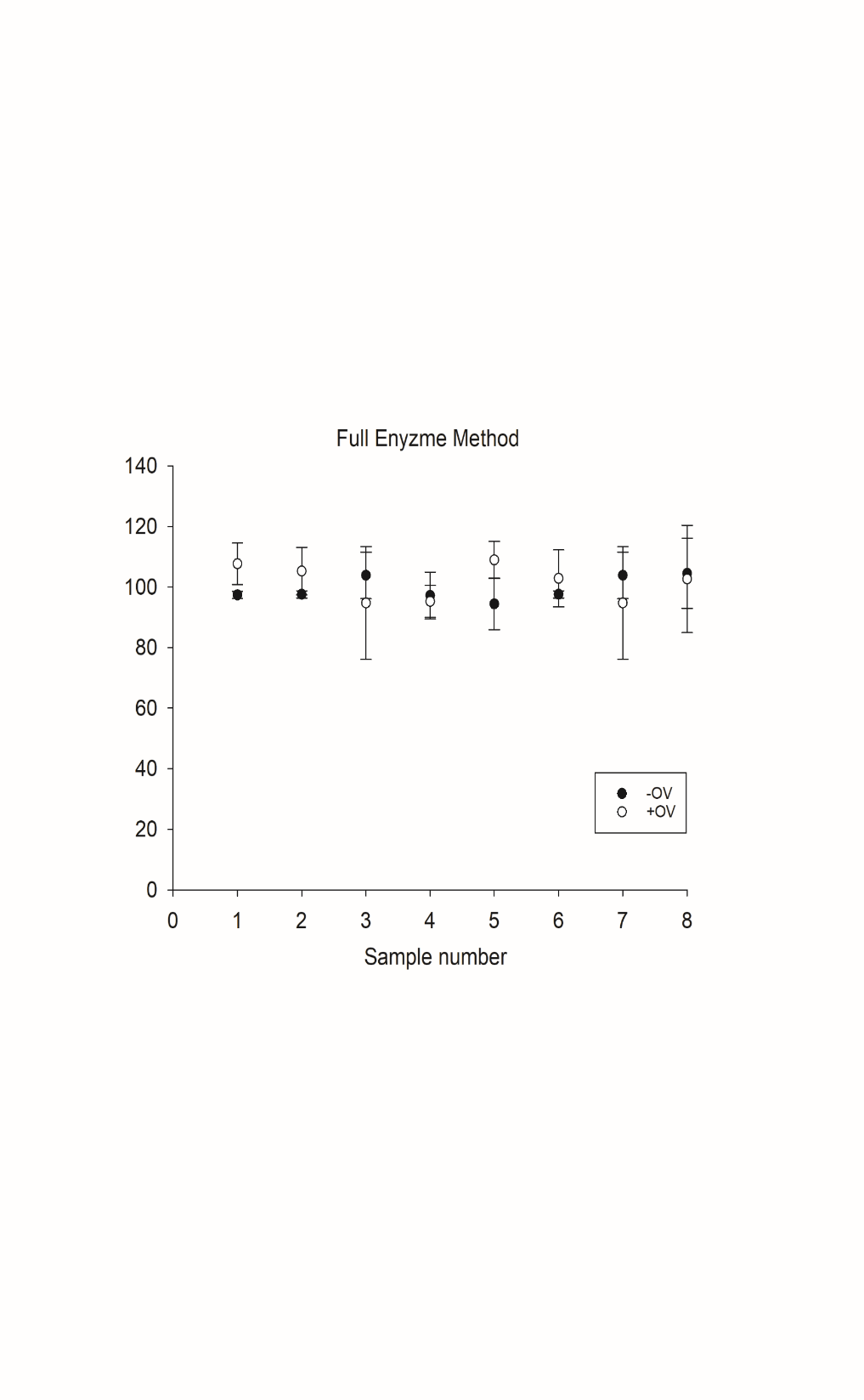

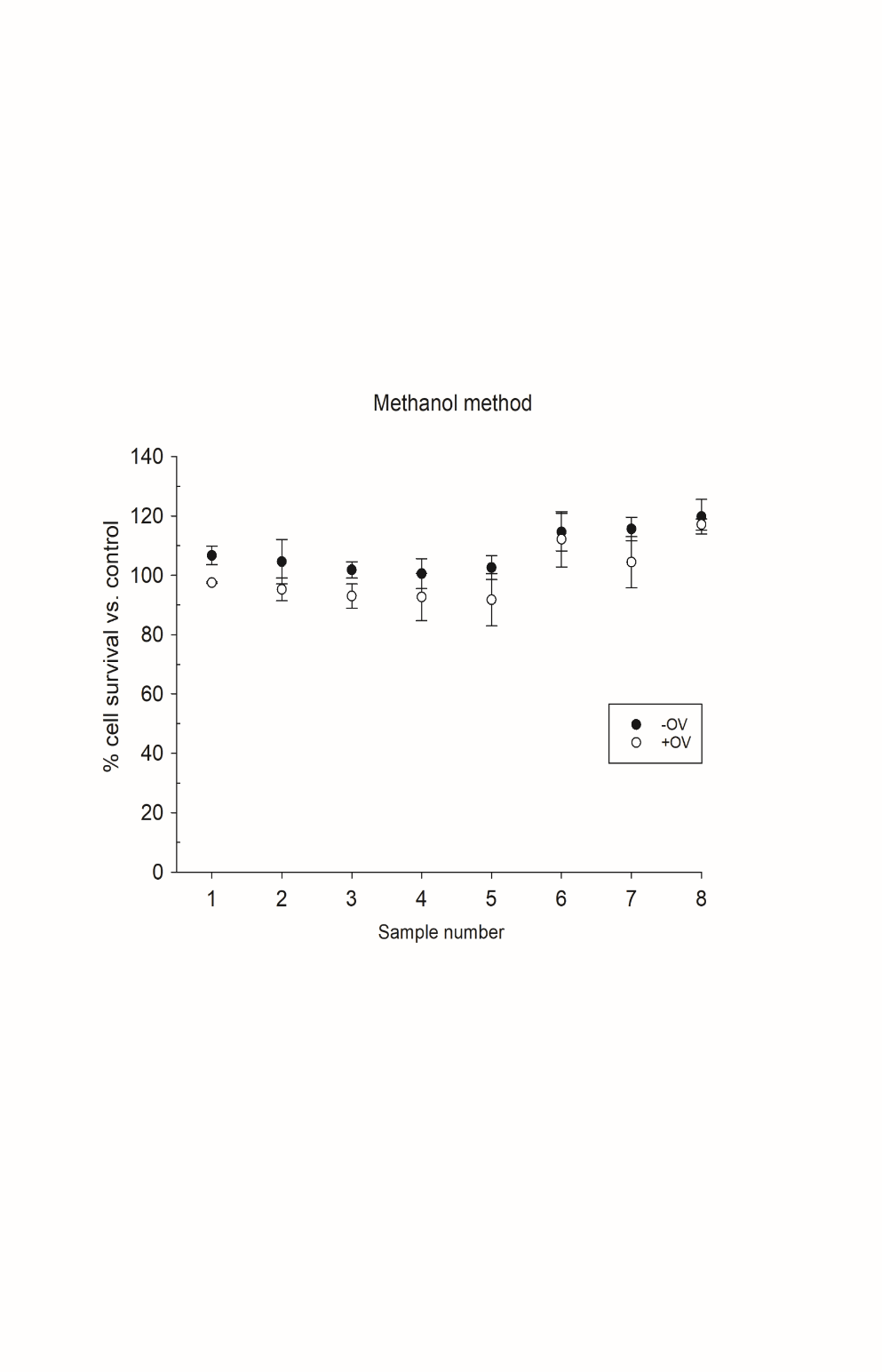


A

B

C

D

Supplemental Figure 1. Matrix effect test using the CBA for each extraction method A) Acetone, B) Methanol, C) Quick-Enzyme, and D)Full-Enzyme . Sample numbers represent eight individual fish samples extracted and analyzed by the CBA after the addition of 10 mg DTE or WTE.





Supplemental Figure 2. LC-MS/MS chromatograms of full clean-up extracts of *E. canina* initially prepared by methods A) Acetone, B) Methanol, C) Quick enzyme, and D) Full enzyme; colored lines show the extracted ion chromatograms of the sodium adducts ([M+Na]^+^) of CTX-1B (1, pink), 2,3-dihydroxyCTX-3C (3, red) and it 49-epimer (2, red), 51-hydroxyCTX-3C (4, blue), 54-deoxyCTX-1B (6, black) and it 52-epimer (5, black), and CTX-3C (8, green) and its 49-epimer (7, green); Panels A, B, and C represent 1 g DTE per mL, panel D 2 g DTE per mL.





Supplemental Figure 3. LC-MS/MS chromatograms of full clean-up extracts of *Lutjanus* spp. initially prepared by methods A) Acetone, B) Methanol, C) Quick enzyme, and D) Full enzyme; colored lines show the extracted ion chromatograms of the sodium adducts ([M+Na]^+^) of 2,3,51-trihydroxyCTX-3C (1, turquoise), 2,3-dihydroxyCTX-3C (3, red) and it 49-epimer (2, red), 51-hydroxyCTX-3C (4, blue), mono-hydroxy-CTX-3C #1 (putative M-*seco*-CTX-3C) (6, grey) and it 49-epimer (5, grey), and mono-hydroxy-CTX-3C #2 (putative 2-hydroxyCTX-3C) (8, grey) and its 49-epimer (7, grey); Panels A, B, and C represent 5 g WTE per mL, panel D 10 g WTE per mL.





Supplemental Figure 4. LC-MS/MS chromatograms of full clean-up extracts of *E. canina* initially prepared by the Acetone method with panels A) to C) showing the results for samples 1 to 3; sample 3 gave a negative result both in the CBA and by LC-MS/MS; panel D) shows the overlay of the extracted ion chromatograms of the sodium adducts ([M+Na]^+^) of CTX-3C and its 49-epimer in samples 1 and 2 (grey lines) compared to sample 3 (black line).
